# Are preoperative phase-contrast CSF flow parameters ideal for predicting the outcome of shunt surgery in patients with idiopathic normal pressure hydrocephalus?

**DOI:** 10.3389/fneur.2022.959450

**Published:** 2022-09-27

**Authors:** Wen-Jie He, Xie-jun Zhang, Qi-Zhong Xu, Run-tao Bai, Jia-kuan Chen, Xi Zhou, Jun Xia

**Affiliations:** ^1^Department of Radiology, The First Affiliated Hospital of Shenzhen University, Shenzhen Second People's Hospital, Shenzhen University, Shenzhen, China; ^2^Department of Neurosurgery, The First Affiliated Hospital of Shenzhen University, Shenzhen Second People's Hospital, Shenzhen University, Shenzhen, China; ^3^Department of Neurology, The First Affiliated Hospital of Shenzhen University, Shenzhen Second People's Hospital, Shenzhen University, Shenzhen, China

**Keywords:** idiopathic normal pressure hydrocephalus, magnetic resonance imaging, phase-contrast MR, ventriculoperitoneal shunt, prognosis

## Abstract

**Purpose:**

Phase-contrast magnetic resonance (PC-MR) is widely used in patients with idiopathic normal pressure hydrocephalus (iNPH), but its role in predicting prognosis remains controversial. To evaluate the effectiveness of preoperative PC-MR CSF flow measurement in predicting the clinical response to shunt surgery in patients with iNPH.

**Methods:**

Forty-six patients with definite iNPH were included between January 2018 and January 2022. PC-MR was used to evaluate CSF peak velocity (PV), average velocity, aqueductal stroke volume (ASV), net ASV, and net flow. The modified Rankin Scale (mRS), iNPH grading scale (iNPHGS), Mini-Mental State Examination (MMSE), and Timed 3-m Up and Go Test (TUG) were used for clinical assessment. The primary endpoint was the improvement in the mRS score 1 year after surgery, and the secondary endpoints were the iNPHGS, MMSE, and TUG scores at 1 year. Differences between shunt improvement and non-improvement groups, based on the clinical outcomes, were compared using the Mann-Whitney U-test, logistic regression models, and receiver operating characteristic curves. Correlations between CSF flow parameters and the baseline clinical outcomes were assessed using Spearman's correlation coefficient.

**Results:**

No CSF parameters significantly differed between shunt improvement and non-improvement groups based on mRS and secondary outcomes. And all CSF parameters showed significant overlap in both shunt improvement and non-improvement groups based on mRS and secondary outcomes. Significant correlations between the mRS and iNPHGS scores, and PV, ASV, and net ASV were observed.

**Conclusion:**

While some preoperative PC-MR CSF flow parameters reflected the symptom severity of iNPH to a certain extent, they alone might not be ideal markers of shunt responsiveness.

## Introduction

Idiopathic normal pressure hydrocephalus (iNPH) is an increasingly recognized treatable disease among older adults and is characterized by a triad of gait impairment, cognitive disturbances, and urinary incontinence, as well as the presence of normal cerebrospinal fluid (CSF) pressure on lumbar puncture (1, 2). Symptom improvement can be achieved after CSF shunting in 50–80% of patients, and the effectiveness varies from patient to patient (3, 4). The survival of iNPH patients with improved CSF shunts is similar to that of the general population, indicating that shunt surgery can, besides improving symptoms and signs, can normalize the survival of such patients (5). Therefore, predictive markers of surgical outcomes are necessary for decision-making regarding surgical indications.

CSF tap tests have been widely used to predict shunt responses in clinical practice. Meanwhile, the infusion test, as one of the useful diagnosis procedure for iNPH, have been also used to predict shunt responses in some clinical centers (6, 7). However, both CSF tap tests and infusion tests have some shortcomings, such as invasiveness and inconsistent predictive ability, with sensitivity and specificity of 42–93 and 20–100%, respectively (6–8). Many studies have attempted to identify non-invasive imaging markers that can predict postoperative outcomes (9). Among them, the predictive value of phase-contrast magnetic resonance (PC-MR), one of the most widely used noninvasive techniques in iNPH patients, has been analyzed in previous studies (9–11). PC-MR was initially reported as a predictive marker of postoperative outcomes in patients with iNPH (11). However, subsequent studies produced conflicting and inconsistent results (9, 12), and the predictive value of PC-MR remains controversial (13). These inconsistencies may stem from differences in the MR equipment used, CSF parameters collected, and the non-standardized clinical protocols of previous studies (9, 10). In addition, some previous studies did not focus on iNPH but mixed it with other types of hydrocephalus, such as chronic adult hydrocephalus (9).

Therefore, in this study, we used consistent MR scanning equipment and PC-MR scanning methods, collected multiple PC-MR CSF parameters, and focused on definite iNPH patients. The purpose of this study was to investigate whether PC-MR measurements of CSF parameters could be used as predictive markers of shunt outcomes in iNPH patients.

## Materials and methods

### Study participants

**Figure 1:**
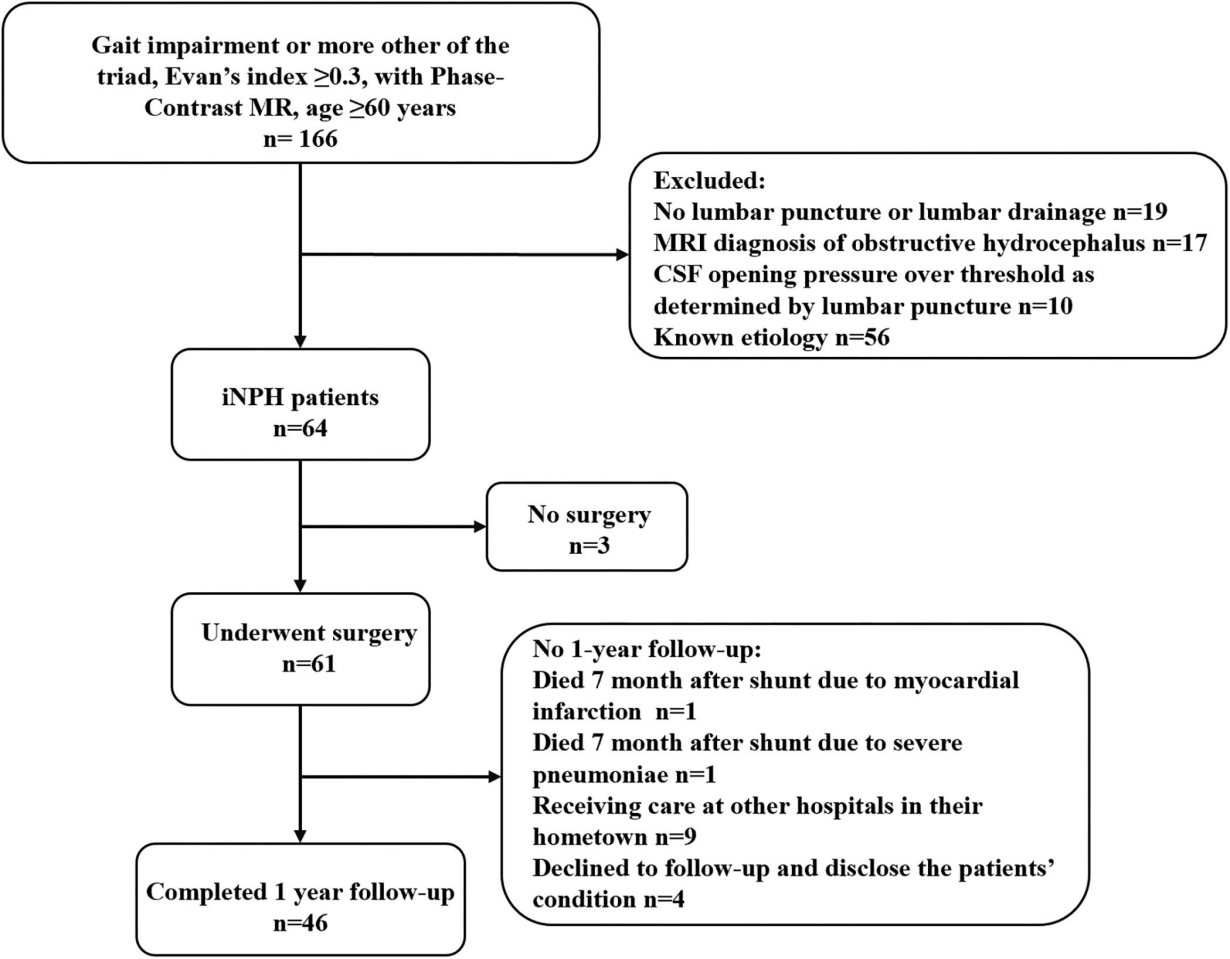
Study flowchart for the inclusion of patients with iNPH from the initial screening to the final analysis. iNPH, idiopathic normal pressure hydrocephalus; MR, magnetic resonance; MRI, magnetic resonance imaging; CSF, cerebrospinal fluid.

The original cohort consisted of 61 consecutive iNPH patients who had undergone PC-MR within 1 week before diagnostic lumbar CSF puncture and received a shunt at the authors' hospital between January 2018 and January 2022. Figure 1 depicts the flow chart for the inclusion of patients with iNPH from the initial screening to the final analysis. Fifteen patients had been lost at the 12-months follow-up visit: one patient died 7 months after shunting due to myocardial infarction, one patient died 6 months after shunting due to severe pneumonia, nine patients were receiving care at other hospitals in their hometown, and four patient's family member declined follow-up and declined to disclose the patient's condition. The final cohort consisted of 46 patients. During the follow-up period, none of these patients developed shunt complications, such as subdural hematomas, shunt infection, or proximal or distal catheter failure.

The inclusion criteria were as follows: (1) meeting the diagnostic criteria of the iNPH guidelines (1, 2), age ≥60 years, presence of one or more symptoms of the triad, ventricular enlargement, no disorders causing ventriculomegaly, and normal CSF pressure on lumbar puncture; (2) PC-MR within 1 week before diagnostic lumbar CSF puncture; (3) a positive response on the CSF tap tests; (4) and same clinical evaluations of the triad before and at 12 months after shunt surgery. The exclusion criteria were: (1) presence of suspected cognitive disorders or unexplained gait disturbances for over 2 years, which would make it difficult to evaluate the clinical symptoms; (2) <1-year of follow-up. Because of the controversy regarding the role of PC-MR, CSF parameters derived from PC-MR have not been used for decision-making regarding shunt surgery at our institution. The protocol was approved by our hospital's bioethics committee (approval no. KS20190114001).

### Clinical assessment and outcomes

Based on both the iNPH guidelines and previous studies (1, 2, 14), the clinical assessments performed included the Mini-Mental State Examination (MMSE), Timed Up and Go Test (TUG), iNPH grading scale (iNPHGS), and modified Rankin Scale (mRS). All patients were prospectively assessed according to the above-mentioned standardized protocols before surgery and at the 12 months postoperative follow-up by a multidisciplinary team specialized in hydrocephalus.

Shunt improvement was defined as an improvement of 1 or more grades on the mRS, ≥1 point on the iNPHGS, and ≥3 points on the MMSE; and a decrease of >10% on the TUG. The primary endpoint was an improvement of ≥1 point (favorable outcome) on the mRS at 12 months after shunt placement. The secondary outcome measures were the iNPHGS, MMSE, and TUG findings.

### MRI sequence

All patients underwent PC-MRI examinations using the same scanning sequence. To reduce heterogeneities in baseline aqueductal flow in PC-MR as much as possible, the scans of all patients with iNPH were performed at night (8–10 pm) (15). All images were obtained using a 3.0T MRI scanner (Prisma, Siemens, Erlangen, Germany) with 20-channel phase-array head coils. All patients underwent 3D-T1 weighted imaging (T1WI) and a retrospective cardiac-gated phase-contrast CSF flow quantification sequence. Sagittal 3D-T1WI scans were performed with a magnetization-prepared rapid acquisition gradient-echo sequence that covered the entire head. The sequence parameters were as follows: repetition time/echo time (TR/TE) = 2,300/2/0.98 ms; flip angle = 9°; slice thickness = 1 mm; field of view, 256 × 256 mm; matrix, 256 × 256; and pixel size, 1 × 1 mm. The acquisition parameters for PC-MRI with peripheral gating were as follows: TR/TE = 21/7 ms; field of view, 160 × 160 mm; matrix, 256 × 205; voxel size, 0.6 × 0.6 × 6.0 mm; slice thickness, 6 mm; distance factor 20%; calculated phases, 40; velocity encoding, 20 cm/s, which was increased to 25 cm/s if aliasing occurred; acquisition time, ~183 s; and flip angle, 10°. In the transverse acquisition plane, the encoding of the flow direction was performed from the feet to the head. And the transverse acquisition plane has been selected to be perpendicular to the aqueduct in the middle of its length, and the phase-correction technique provided by the vendor was applied. During diastole, CSF flows in the velocity-encoded cine direction from the feet to the head, indicating forward flow. During systole, CSF flows in the velocity-encoded cine direction from the head to the feet, indicating a backward flow. Positive values represent the caudocranial direction, and negative values represent the craniocaudal direction (15).

### Imaging analysis

**Figure 2:**
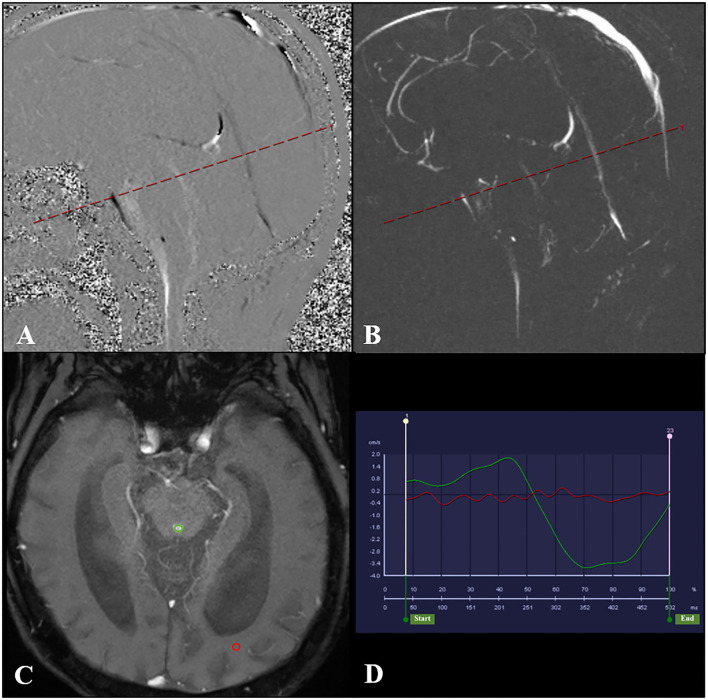
PC-MR was performed with the slice orientation perpendicular to the aqueduct. **(A,B)** The aqueductal flow curve is based on a section orientation (red line) perpendicular to the aqueduct. And the transverse acquisition plane (red line) has been selected to be perpendicular to the aqueduct in the middle of its length. **(C)** PC-MR with a manually drawn region of interest (green circle) defining the aqueduct. The reference region of interest is shown by the red circle. **(D)** The red line represents the flow of the reference region of interest, and the green line represents the CSF flow through the aqueduct. PC-MR, phase contrast-magnetic resonance; CSF, cerebrospinal fluid.

Quantitative analysis of CSF flow parameters was performed using the flow quantification software provided with the MR scanner. The region of interest was manually defined along the outer border of the aqueduct by the unified opinions of three neuroradiologists with more than 15 years of experience in brain MRI interpretation to reduce the difference in measurement (Figure 2). CSF flow in the craniocaudal direction was defined as antegrade flow. The peak velocity (PV), average velocity (AV), average flow over range, aqueductal stroke volume (ASV), net ASV, and net flow were measured. ASV was defined as the mean volume of CSF flowing craniocaudally during systole and caudocranially during diastole (11, 16, 17).

The net ASV was calculated by subtracting the PC-MRI-derived volumetric estimate of retrograde aqueductal flow from that of antegrade flow during one cardiac cycle (18, 19). Net flow (mL/min) represents the net CSF aqueductal flow rate per minute and was calculated by multiplying the net ASV by the heart rate, which was measured using the finger pulse trigger of the MRI scanner (16, 20).

### Statistical analysis

All statistical analyses were performed using the SPSS version 24 (IBM Corporation, Armonk, NY, USA). The Shapiro-Wilk test of normality was used to determine the distribution of variables. A *t-*test was used to identify significant differences among normally distributed data between the improvement and non-improvement groups, such as age. Similarly, the Mann-Whitney and chi-square tests (when appropriate) were used to test for differences in CSF flow parameters between shunt responders and non-responders. Associations between outcome and CSF flow parameters were assessed using logistic regression models, with results presented as odds ratios with 95% confidence intervals (CIs) in a forest plot. Receiver operating characteristic curves (ROCs) were used to evaluate the predictive effectiveness of the CSF flow parameters on the results. Correlations between the CSF flow parameters from PC-MR and clinical assessment parameters were determined using the Spearman correlation coefficient. Statistical significance was accepted at the level of 0.05 (two-tailed).

## Results

The preoperative demographic characteristics, preoperative clinical characteristics, and PC-MR CSF flow results of the iNPH patients are summarized in Table 1. The 46 patients with iNPH consisted of 24 men and 22 women, with a median age at the time of shunt surgery of 70.4 ± 5.7 years (range: 61–80 years). The mean time to follow-up was 61 ± 6 weeks after surgery. The average duration of symptoms was 2.3 years (range: 0.5–7 years). According to preoperative symptom evaluations, 46 patients had gait disturbance, 34 had cognitive impairment, 22 had urinary symptoms, and 22 had the classic triad.

**Table 1:** Demographic information, preoperative clinical characteristics, and PC-MR CSF flow parameters in 46 patients with iNPH.

**Characteristic**	**Value**
**Demographic information**
Age (years)	70.5 ± 5.9
Gender (female/male)	22/24
Duration of symptoms (months)	12.0 (18.0)
Mean CSF pressure (cm/H_2_O)	151 ± 27
**Preoperative clinical outcomes**
mRS score	2.57 ± 1.12
iNPHGS score	5.87 ± 2.70
MMSE score	20.5 (6.0)
TUG (second)	29.1 (18.2)
Triad (N)	22
Gait Impairment + Cognitive Disturbances (N)	12
Gait Impairment + Urinary Incontinence (N)	0
Urinary Incontinence + Cognitive Disturbances (N)	0
Gait Impairment Only (N)	12
Cognitive Disturbances Only (N)	0
Urinary Incontinence Only (N)	0
**PC-MR CSF flow**
ASV (mL)	0.0135 (0.071)
net ASV (mL)	0.006 (0.017)
Average flow over range (mL/s)	0.007 (0.020)
Average velocity (cm/s)	0.207 (0.346)
Peak velocity (cm/s)	4.36 (8.22)
Net flow volume (mL)	0.552 (1.218)

iNPH, idiopathic normal pressure hydrocephalus; ASV, aqueductal stroke volume; MMSE, Mini-Mental State Examination; mRS, modified Rankin Scale; TUG, Timed Up and Go Test; iNPHGS, iNPH grading scale. Nonparametric data are given as median (interquartile range).

One year after surgery, 28 patients showed improved mRS scores and iNPHGS scores. Twenty-five (74%) patients showed improvement on the MMSE by at least three levels (definitive improvement in cognition). Twenty-eight (61%) of the 46 patients tested exhibited a >10% decrease in the TUG, and 10 (45%) showed improved continence.

Patients with iNPH were divided into shunt improvement and non-improvement groups, based on the preoperative and postoperative mRS scores, and their PC-MR CSF parameters and preoperative clinical characteristics were compared (Table 2). We did not find any significant differences in PC-MR CSF parameters (PV, AV, average flow over range, ASV, net ASV, and net flow) between improvement and non-improvement groups. Similarly, no differences in the preoperative clinical characteristics were observed.

**Table 2:** Preoperative characteristics of patients with and without an improved mRS score at 1 year after surgery.

**Characteristic**	**Improvement** **(n = 28)**	**No Improvement** **(n = 18)**	**p-value^†^**
Demographic information
Age (years)	70.1 ± 6.2	70.9 ± 5.3	0.685
Mean CSF pressure (cm/H^2^O)	140 (45)	155 (56)	0.663
Duration of symptoms (months)	12.0 (24.0)	12.0 (28.0)	0.925
**Preoperative clinical outcomes**
mRS score	2.0 (2.0)	2.0 (2.0)	0.891
iNPHGS score	6.0 (3.0)	5.5 (5)	0.531
MMSE score	21.5 (6.0)	20.0 (7.0)	0.419
TUG (second)	27.1 (16.3)	30.1 (19.7)	0.590
**PC-MR CSF flow**
ASV (mL)	0.0113 (0.0695)	0.0200 (0.0740)	0.162
net ASV (mL)	0.0080 (0.015)	0.0060 (0.017)	0.527
Average flow over range (mL/s)	0.0080 (0.020)	0.0070 (0.016)	0.787
Average velocity (cm/s)	0.243 (0.232)	0.208 (0.381)	0.620
Peak velocity (cm/s)	3.775 (7.354)	5.937 (10.025)	0.316
Net flow volume (mL)	0.666 (1.192)	0.0.528 (1.316)	0.787

iNPH, idiopathic normal pressure hydrocephalus; ASV, aqueductal stroke volume; MMSE, Mini-Mental State Examination; mRS, modified Rankin Scale; TUG, Timed Up and Go Test; iNPHGS, iNPH grading scale. Nonparametric data are given as median (interquartile range).^†^significance determined by the Mann-Whitney U-test.

**Figure 3:**
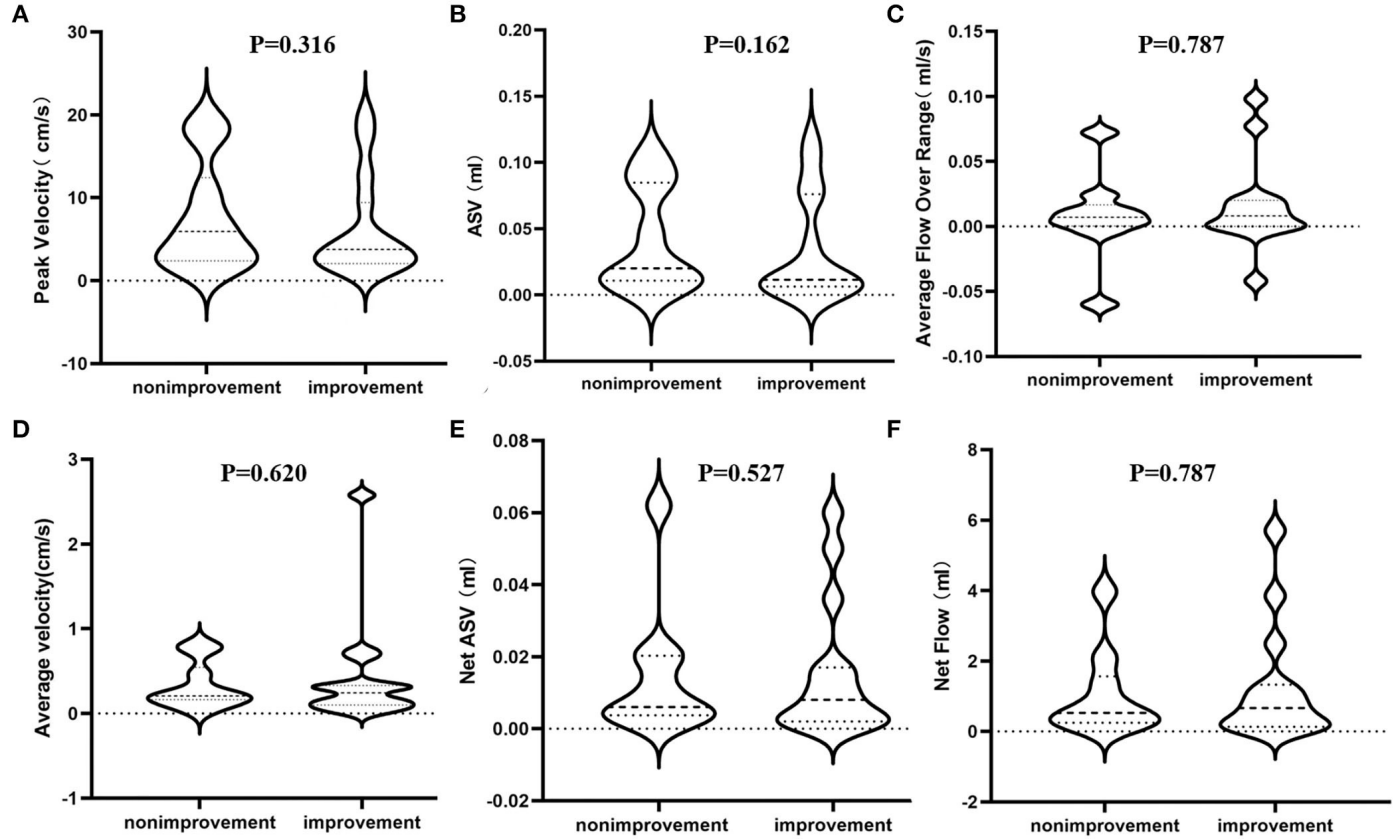
Differences in estimated PC-MR CSF flow parameters between shunt improvement and non- improvement groups based on the mRS scores. **(A)** The peak velocity in the aqueduct; **(B)** the ASV in the aqueduct; **(C)** the average flow over the range in the aqueduct; **(D)** the average velocity in the aqueduct; **(E)** the net ASV in the aqueduct; **(F)** the net flow in the aqueduct. PC-MR, phase contrast-magnetic resonance; ASV, aqueductal stroke volume; mRS, modified Rankin Scale.

Subsequently, we divided iNPH patients into shunt improvement and non-improvement groups based on the secondary outcome measures (iNPHGS scores, MMSE scores, and TUG) before and after shunt surgery, and compared their PC-MR CSF parameters (Table 3). Similarly, we found that all CSF parameters were not significantly different between improvement and non-improvement groups according to all secondary outcomes. There were also no significant differences in preoperative clinical characteristics. In addition, regardless of the mRS and secondary outcome measures, we found that all CSF parameters showed significant overlap among shunt responders and non-responders (Figure 3).

**Table 3:** Preoperative characteristics of patients with and without improved secondary outcomes at 1 year after surgery.

**Characteristic**	**iNPHGS**			**TUG**			**MMSE**		
	**Improvement**	**No improvement**	***p*-value^†^**	**Improvement**	**No improvement**	***p*-value^†^**	**Improvement**	**No improvement**	***p*-value^†^**
**Demographic information**
Age (years)	69.7 ± 6.1	71.6 ± 5.1	0.410	69.6 ± 6.7	71.0 ± 5.2	0.526	68.9 ± 6.3	71.2 ± 5.4	0.284
Mean CSF pressure (cm/H_2_O)	140 (38)	160 (40)	0.383	140 (43)	150 (51)	0.448	138 (33)	150 (30)	0.184
Duration of symptoms	12 (21)	12 (32)	0.677	12 (42)	12 (19)	0.974	10 (15)	12 (55)	0.359
**Preoperative clinical outcomes**
mRS score	2.0 (2.0)	2.0 (2.0)	0.383	2.0 (2.0)	2.0 (1.0)	0.834	2.0 (2.0)	2.0 (1.0)	0.411
iNPHGS score	6.5 (4.0)	5.0 (5.0)	0.192	7.0 (3.0)	5.5 (5.0)	0.445	6.5 (4.0)	6.0 (4.0)	0.282
MMSE score	21.0 (6.0)	19.0 (7.0)	0.123	20.5 (6.0)	21.5 (6.0)	0.732	19.5 (6.0)	21.5 (7.0)	0.143
TUG (second)	26.6 (16.7)	31.4 (18.7)	0.218	26.3 (15.1)	30.5 (18.5)	0.132	28.6 (15.3)	32.3 (18.4)	0.183
**PC-MR CSF flow**
ASV (mL)	0.011 (0.070)	0.020 (0.076)	0.329	0.016 (0.072)	0.013 (0.076)	0.659	0.011 (0.088)	0.016 (0.071)	0.872
net ASV (mL)	0.008 (0.020)	0.006 (0.017)	0.659	0.006 (0.033)	0.008 (0.017)	0.752	0.011 (0.030)	0.006 (0.017)	0.923
Average flow over range (mL/s)	0.008 (0.021)	0.007 (0.021)	0.850	0.009 (0.050)	0.005 (0.020)	0.298	0.007 (0.023)	0.007 (0.021)	0.722
Average velocity (cm/s)	0.243 (0.324)	0.207 (0.481)	0,753	0.127 (0.268)	0.224 (0.516)	0.257	0.244 (0.502)	0.207 (0.353)	0.897
Peak velocity (cm/s)	3.725 (8.214)	5.886 (11.813)	0.528	4.310 (13.118)	3.980 (7.26)	0.801	3.797 (9.817)	4.310 (8.174)	0.948
Net flow volume (mL)	0.666 (1.496)	0.528 (1.521)	0.877	0.552 (2.513)	0.654 (1.316)	0.850	0.847 (2.025)	0.528 (1.218)	0.996

iNPH, idiopathic normal pressure hydrocephalus; ASV, aqueductal stroke volume; MMSE, Mini-Mental State Examination; mRS, modified Rankin Scale; TUG, Timed Up and Go Test; iNPHGS, iNPH grading scale. Nonparametric data are given as median (interquartile range).^†^Significance determined by the Mann-Whitney U-test.

**Figure 4:**
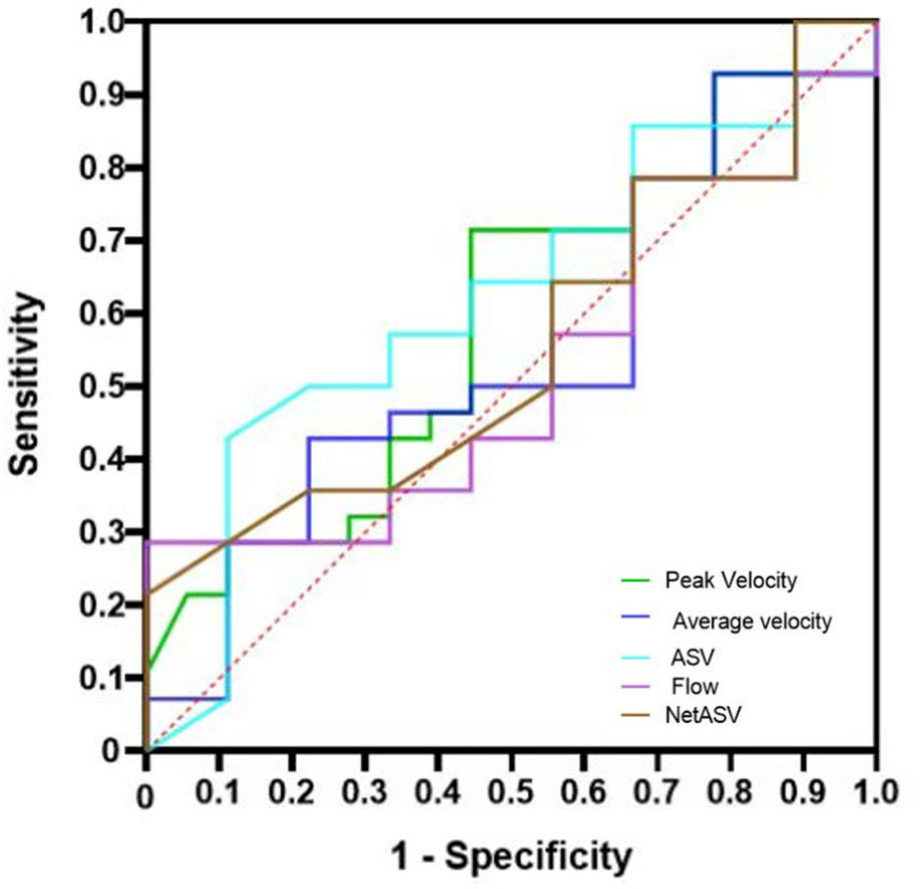
ROC and AUC of the mRS scores to differentiate the improvement group from the non-improvement group. ROC: receiver operating curve, AUC: area under the curve; mRS, modified Rankin Scale.

**Figure 5:**
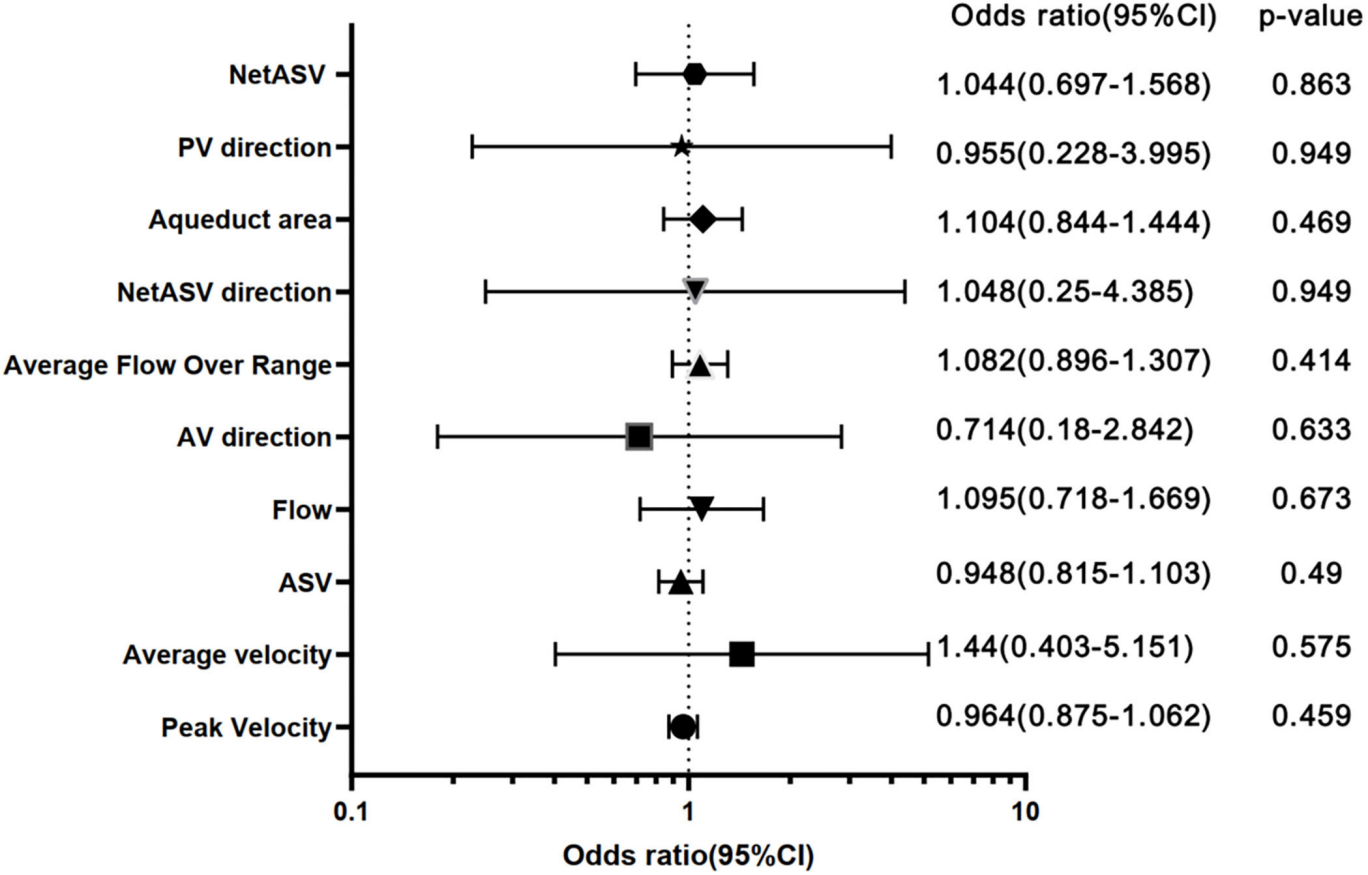
Forest plot with sex- and age-adjusted odds ratios for all imaging features. ORs with a 95% CI of 1-SD increase for continuous variables and a 1-U increase for dichotomous and ordinal variables are shown. ASV, aqueductal stroke volume; PV direction, peak velocity direction; AV direction, average velocity.

In addition, using different outcome indicators, the ROC analysis of PC-MR CSF parameters showed no significant difference between groups (*p* > 0.05). Under the primary result, the AUCs of the PC-MR CSF parameters were 0.58 (95% CI: 0.42–0.76, *p* = 0.317) for peak velocity, 0.54 (95% CI: 0.37–0.72, *p* = 0.621) for average velocity, 0.62 (95% CI: 0.45–0.79, *p* = 0.177) for ASV, 0.56 (95% CI: 0.39–0.72, *p* = 0.539) for net ASV and 0.52 (95% CI: 0.35–0.69, *p* = 0.787) for net flow, respectively (Figure 4). In conclusion, the diagnostic performance for the treatment response of PC-MR CSF parameters was poor and not significant, further corroborating our previous results. Binary logistic regression analysis was performed on all imaging indexes included in the two scores with the primary result as the classification standard. The predicted values are expressed after adjustment for sex and age (Figure 5). No PC-MR CSF parameters were significantly associated with postoperative improvement.

Furthermore, we analyzed the relationship between PC-MR CSF flow parameters and the clinical outcomes of patients with iNPH (Table 4). There were significant correlations between the mRS and iNPHGS scores, and several PC-MR parameters (PV, ASV, and net ASV). However, there were no associations between the duration of symptoms or any preoperative clinical characteristics and PC-MR CSF flow parameters.

**Table 4:** Relationship between PC-MR CSF flow parameters and clinical outcomes in iNPH patients.

**Characteristic**	**mRS**	**iNPHGS**	**TUG**	**MMSE**
Duration of symptoms	0.003 (0.998)	0.019 (0.932)	0.127 (0.553)	0.131 (0.550)
ASV	−0.578 (0.004) *	−0.586 (0.003) *	−0.327 (0.101)	−0.297 (0.216)
net ASV	−0.434 (0.039) *	−0.463 (0.026) *	−0.504 (0.014)	−0.346 (0.106)
Average flow over range	−0.401 (0.058)	−0.322 (0.134)	−0.216 (0.321)	−0.314 (0.145)
Average velocity	−0.046 (0.835)	−0.127 (0.564)	−0.275 (0.122)	0.046 (0.834)
Peak velocity	−0.452 (0.030) *	−0.426 (0.043) *	−0.350 (−0.131)	−0.314 (0.144)
Net flow volume	−0.362 (0.090)	−0.406 (0.055)	−0.278 (0.181)	−0.282 (0.192)

ASV, aqueductal stroke volume; MMSE, Mini–Mental State Examination; mRS, modified Rankin Scale; TUG, Timed Up and Go Test; iNPHGS, iNPH grading scale. ^*^ A significant relationship according to the Spearman correlation coefficient.

## Discussion

The main observation of this study was that none of the PC-MR CSF parameters differed significantly between shunt improvement and non-improvement groups among patients with iNPH. Meanwhile, there were some associations observed between preoperative PC-MR CSF flow parameters and clinical outcomes in patients with iNPH.

Although PC-MR-based aqueductal assessment of CSF hydrodynamics has previously been extensively investigated and advocated as a tool for the selection and prognostication of iNPH patients for shunt surgery (13), its value is still controversial (18). Previous studies showed significant heterogeneity due to continuously evolving iterations of MR technology and differences in PC-MR parameters (9). Most prior studies lacked multiple PC-MR parameters and only assessed a single variable, such as ASV, net ASV, or flow rate, leaving the possibility of the conclusion changing with other variables (9, 16, 17). Increasing evidence shows that the measurement of PC-MR CSF parameters is machine- and software-dependent, involving factors such as image resolution, pixel size, and MRI field strength (18, 19, 21). In addition, in previous studies, some of the subjects did not meet the diagnostic criteria stipulated in the Japanese and international iNPH guidelines (1, 2), and acquired hydrocephalus patients may have been included (9). Furthermore, a recent publication proposed that each treatment center should determine “normal” PC-MR parameters for the scanner by examining a number of healthy elderly individuals (18). Therefore, the strengths of this study were the consistent use of a 3.0 T MR machine, appropriate PC-MR scan parameters, and evaluation of multiple CSF parameters based on previous studies to avoid controversies caused by inconsistencies in PC-MR and reduce the impact of PC-MR differences.

The efficiency of shunt surgery in our study (61%) was comparable to that in previous studies (9, 22). After minimizing differences in MRI equipment and PC-MR scan parameters, we did not find significant differences in any PC-MR CSF parameters between CSF shunt improvement and non- improvement groups, based on either primary or secondary outcomes. This is different from some previous studies, which demonstrated that patients with iNPH who have high-velocity aqueductal flow on MRI were more likely to respond to shunting (13). However, the shunt surgery results were inconsistent in iNPH patients with the high aqueductal flow in this study. Furthermore, we found that all CSF parameters in the shunt responders and non-responders significantly overlapped, as reported in previous studies (9, 23). In fact, the concept of PC-MR is based on a single “snapshot” of aqueductal flow characteristics, representing a short period of CSF flow. Blitz et al. reported significant heterogeneities even in baseline PC-MR aqueductal flow parameters in the normal state (9). For example, ASV, one of the most frequently mentioned and the most important parameter for diagnosing and predicting the prognosis of iNPH using PC-MR, generally behaved as a dynamic variable in previous studies, increasing in the early phase but decreasing after a peak later in the disease course, potentially due to brain ischemia (24). Moreover, ASV can be easily affected by several factors, such as cross-sectional aqueductal area, ventricle volume, and heart rate (9, 21, 23). Therefore, due to the variability of ASV, the value of quantifying specific ASV values to assess iNPH prognosis is uncertain.

In addition to ASV, net ASV, which was used to diagnose iNPH, is another frequently noted PC-MR parameter. However, measurements of the net ASV are associated with technical difficulties similar to those of ASV (10, 17). The value of the net ASV is very small, in the order of microliters, and is calculated from the significantly larger bidirectional flow (10, 25). It only takes small variations in the bidirectional flow to lead to imprecise estimations of the net ASV (10, 25).

In addition to PC-MR parameter measurements, which easily vary, intraventricular CSF flow measurements in iNPH patients also show substantial variations. Jaeger et al. found that substantial variations in the mean wave amplitudes of iNPH patients over the course of several hours of intracranial pressure (ICP) monitoring are common, demonstrating that intracranial pulsatility is not stable, but rather a highly fluctuating phenomenon (19). These variations might represent physiological fluctuations, similar to physiological slow wave activity in the ICP signal (19). These physiological fluctuations might obscure the MRI snapshot measures of intracranial pulsations. Based on the characteristics of 24-h CSF fluctuation in iNPH patients in 24 h, and the specific conditions at the hospital, we selected 8–10 pm as the scanning time of PC-MR to reduce the effects of CSF fluctuation as much as possible (15). Even so, none of the preoperative PC-MR parameters were associated with clinical improvement after shunt placement in patients with iNPH in our study. PC-MR parameters only reflect the instantaneity of CSF flow within several minutes of scanning, and the values in an individual patient during this short investigation are not necessarily generalizable to those occurring over 24 h in the same patient.

Moreover, CSF flow itself undergoes dynamic changes, which increases the variability of PC-MR parameters over time, casting further doubt over the value of this method as a prognostic marker. Therefore, we believe that it is difficult to determine the prognosis of iNPH patients post-shunting using only aqueductal CSF parameters in PC-MR. Accordingly, our results question the clinical utility of PC-MR parameters, both with respect to its ability to diagnose iNPH and its value in predicting a clinically favorable shunt response.

Furthermore, we found negative correlations between some PC-MR parameters and clinical symptoms, where the more severe the symptoms of iNPH, the lower the PC-MR CSF dynamics. Previous studies found that patients with iNPH have more severe symptoms with a longer duration (26), and that progressive decline in PC-MR CSF flow parameters occurs in untreated patients with worsening clinical symptoms (24), which could explain the negative correlation between some of the PC-MR parameters and clinical symptoms in our study. History of the symptoms and duration of symptoms in patients with iNPH could be discussed as a potential impact on the shunt outcome (24, 26, 27). However, we did not find a significant correlation between PC-MR parameters and symptom duration, or a clear linear correlation between clinical symptoms and their duration. This may be due to the sample size, which may have been insufficient for definitively identifying correlations. Moreover, previous studies compared the longitudinal changes in patients with iNPH (24), rather than the comparison of PC parameters and symptom duration in this study, which methods lacked comparability.

Our study has some limitations. Although the number of iNPH patients in our study was relatively larger than that in previous PC-MR studies (11, 13), the cohort was nevertheless small, and we were unable to effectively stratify the patients. Second, this study used the standard approach of manual selection of regions of interest (ROIs) rather than automatic acquisition. Automatic acquisition of ROIs may reduce errors (9). However, most institutions still use the traditional manual acquisition method, meaning that our approach is more in line with the actual practices of most hospitals. In addition, we did not assess PC-MR parameters at 12 months postoperatively in most patients due to the long waiting periods for outpatient appointments for PC-MR examination, where many patients were unwilling to wait for such periods. Therefore, we were unable to compare changes in PC-MR CSF flow parameters between postoperative and 12 months postoperative levels, nor the relationship between postoperative CSF parameters and clinical outcomes.

## Conclusion

In conclusion, there were no significant differences in any PC-MR CSF parameters between iNPH patients with and without improvement after shunting. In addition, some PC-MR parameters were associated with symptom severity. Based on these findings, while PC-MR CSF flow parameters may reasonably reflect the severity of iNPH symptoms, used alone, they are not ideal markers for predicting shunt responsiveness in iNPH.

## Data availability statement

The original contributions presented in the study are included in the article/supplementary material, further inquiries can be directed to the corresponding author/s.

## Ethics statement

The studies involving human participants were reviewed and approved by Shenzhen Second People's Hospital Bioethics Committee (Approval No. KS20190114001). The patients/participants provided their written informed consent to participate in this study.

## Author contributions

W-JH performed the experiments, analyzed the data, and wrote the original draft of the manuscript. X-jZ and Q-ZX performed the experiments, analyzed the data, and revised the manuscript. XZ contributed to the experiments. Q-ZX contributed to the data analysis. J-kC contributed to the experiment design and manuscript revision. JX designed the project, supervised the experiments, and drafted and revised the manuscript. All authors read and approved the final manuscript.

## Funding

This work was supported in part by the National Natural Science Foundation of China (Grant No. 82171913) and the Project of Shenzhen International Cooperation Foundation (GJHZ20180926165402083).

## Conflict of interest

The authors declare that the research was conducted in the absence of any commercial or financial relationships that could be construed as a potential conflict of interest.

## Publisher's note

All claims expressed in this article are solely those of the authors and do not necessarily represent those of their affiliated organizations, or those of the publisher, the editors and the reviewers. Any product that may be evaluated in this article, or claim that may be made by its manufacturer, is not guaranteed or endorsed by the publisher.
